# Quality of Care for Prostate Cancer in Kashmir, India: A Real-World Study

**DOI:** 10.7759/cureus.43507

**Published:** 2023-08-15

**Authors:** Omar S Akhtar, Sayed Abdur R Andrabi, Pakeezah S Bhat, Shad S Akhtar

**Affiliations:** 1 Centre of Urology, Hakim Sanaullah Specialist Hospital and Cancer Centre, Sopore, IND; 2 Medical Oncology and Palliative Care, Dr. Shad Salim’s Oncology Centre, Srinagar, IND; 3 Medical Oncology, Dr. Shad Salim’s Oncology Centre, Srinagar, IND; 4 Medical Oncology, Hakim Sanaullah Specialist Hospital and Cancer Centre, Sopore, IND

**Keywords:** prostate care, quality indicators, quality of care, cancer care, prostate cancer

## Abstract

Purpose

Despite the importance of quality care for patients with prostate cancer, significant gaps exist in healthcare delivery, including diagnosis and treatment. Our objective was to assess the quality of care (QoC) using retrospective data from prostate care patients in our center.

Methods

We performed a retrospective study of prostate cancer patients registered at a dedicated cancer care center in the Kashmir region from 2012 to 2020. A set of 15 quality indicators representing crucial facets of diagnosis, pathology, and treatment was identified from a comprehensive list developed and validated by other researchers.

Results

The final analysis of all indicators was conducted on 46 patients with a median age of 70 years (52-92 years). In the majority of patients, the diagnosis (89.1%) was made through a prostatic biopsy, while only five patients were diagnosed solely based on the prostate-specific antigen. Transrectal ultrasound (TRUS) or transurethral resection (TURP)-guided biopsy was documented in 84.8% of patients, with Gleason grading documented in 87.5% of patients. However, the number of positive cores was mentioned for only 25.7% of patients. Radical prostatectomy was the primary treatment for most patients with localized prostate cancer (58.3%). The majority of patients with metastatic prostate cancer were treated with orchidectomy (55%), owing to easy access and the lower cost of surgical castration.

Conclusion

The study demonstrated a lack of compliance with many QoC indicators at the diagnostic and therapeutic levels. However, large-scale, population-based studies are needed to establish the compliance of prostate cancer QoC in Kashmir. The quality indicator assessment can guide the necessary actions required to improve QoC for prostate cancer patients.

## Introduction

Kashmir is a landlocked area in the Himalayas, known as the paradise on Earth. There is scarce information on cancer epidemiology and the burden of cancer in Kashmir. Most of the data available is from single-center hospital-based studies. The most common cancers in Kashmir include the stomach, esophagus, lung, breast, and colorectal cancers [1,2]. Prostate cancer (PC) constitutes around 2.1%-2.5% of all cancers [2,3].

Recently, attention has been paid to the quality of care for cancer patients. Although newer treatments or diagnostic processes are available, evidence suggests that they do not necessarily result in optimum care for cancer patients. Furthermore, increasing data indicates that care for cancer patients may be underused or overdone. Thus, the assessment of the quality of care (QoC) is a matter of concern both for healthcare providers and healthcare recipients [4,5].

QoC studies can typically assess the quality of care provided at the societal level, providing an accurate picture of the regional care pattern, free of sample bias. This could be done using the information from the cancer registries [6]. It is possible to document the quality of treatment delivered and offer regular feedback to healthcare professionals and decision-makers through specific quality indicators (QI), which are used for QoC studies [6]. The QoC can be broadly evaluated based on three categories of QI, viz., structure, process, and outcome indicators [7]. The quality of the technical process is of high significance. It assesses whether the right choices are made in diagnosis and treatment and whether care is provided effectively and skillfully. The process indicators that have been scientifically proven to improve outcomes are the best indicators to assess the quality of care [8].

However, the concept of cancer care quality is becoming broader and incorporates outcome measures, patient preferences, and proper communication between the treating physician and patients. Experts suggest that the delivery of high-quality care for cancer patients is important to ensure optimal outcomes, but it is also challenging to measure [9].

This study aimed to retrospectively assess the results of some process-related QI related to the diagnosis and treatment of PC patients. These patients visited Hakim Sanaullah Specialist Hospital and Cancer Center (HSSHCC), Sopore, or its satellite center in Srinagar, from 2012 to 2020. The QI assessment will serve to evaluate the QoC provided to PC patients at the regional level, in areas with standardized legal, medical, and geographic characteristics.

## Materials and methods

Study population

This retrospective study identified PC patients from the hospital database. Eligible patients were men who were diagnosed with PC from 2012 to 2020 and were registered at Hakim Sanaullah Specialist Hospital and Cancer Center (HSSHCC), Sopore, Kashmir, or its satellite center, Dr. Shad Salim’s Oncology Center in Srinagar, Kashmir, India. The patients were diagnosed or registered for follow-up or palliative care at HSSHCC, Sopore, or the satellite center in Srinagar. The patients registered for follow-up or palliative care had received initial care in other healthcare facilities. At the time of registration at HSSHCC, Sopore, or the satellite center, all such patients were expected to present documentation of their prior clinical contacts, admission information, investigations, and interventions. This retrospective study was approved by the Institutional Ethics Committee of the hospital (Approval No. IEC/2021-02/21.7.21). Patient consent was waived as this was a retrospective chart review and patient data was anonymous.

Data collection

The details of the patients who were included in the study were extracted from their charts. These data included detailed reports of their previous clinical visits, investigations, and interventions.

A set of QI representing crucial facets of the care given to patients diagnosed with PC was used by our research team, encompassing the clinical domains of diagnosis, pathology, and treatment. The QI were selected from a comprehensive list developed and validated by other researchers [6,10,11]. Further, the staging of the disease was registered based on the American Joint Committee on Cancer (AJCC) staging manual (8th edition) [12]. A proforma was developed based on these QIs, and data was extracted from the case records of the patients. Researchers collected the required information directly from the patient’s case records, preventing erroneous interpretations and enabling the uniform codes required to reach a high degree of comparability. All the patient data was anonymized.

A total of 15 indicators were selected for analysis, including five for diagnosis, four for pathology, and six for treatment. All the information needed for these QIs was already present in the medical records of eligible patients. A numerator, the number of patients who met the precise criteria, and a denominator, the number of eligible patients, were used to define each QI. The frequency of each QI was calculated as a percentage by entering the data into an Excel spreadsheet (MS Excel, MS Office 365, Microsoft, Redmond, WA).

## Results

**Figure 1:**
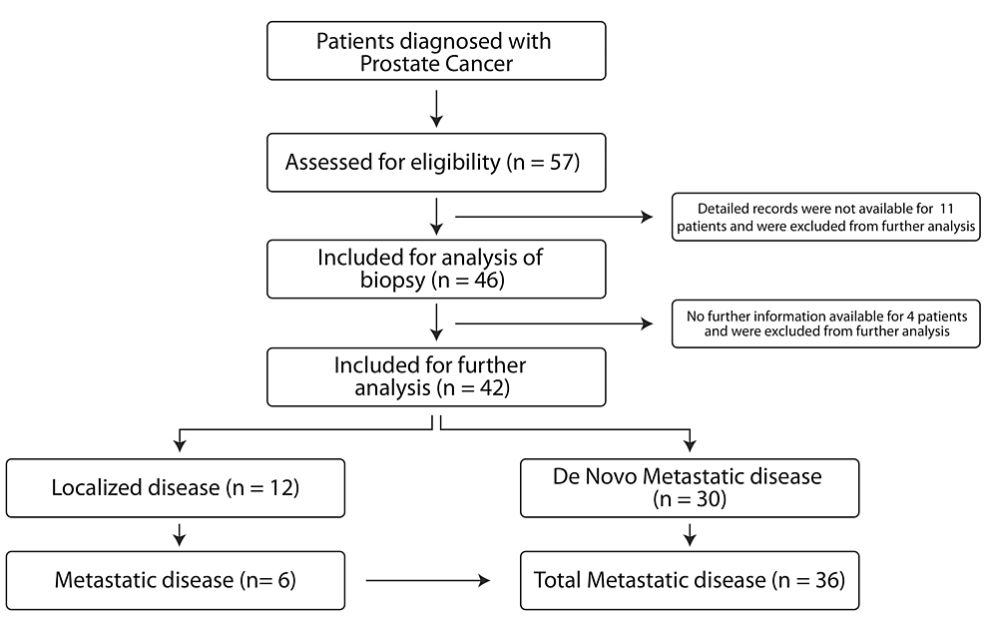
Patient enrollment for analysis

A total of 57 patients were identified who were registered for PC between 2012 and 2020. The demographic details of these patients are described in Table 1. The average age of the patients was 70 ± 9.6 years (mean ± standard deviation). Most of the patients lived in urban areas (37/57, 64.9%). However, since detailed records for 11 patients were not available, they were excluded from further analysis. The final analysis included data from 46 patients (Figure 1).

**Table 1:** Characteristics of registered prostate cancer patients ADT: androgen deprivation therapy; CAB: complete androgen blockade; LHRH: luteinizing hormone-releasing hormone; SD: standard deviation

Parameter	Characteristic	Number of patients n (%)
Age	Mean (±SD) (range)	70 years (±9.6) (52-92 years)
Residence (n=57)	Rural	37 (64.9)
	Urban	20 (35.1)
Site of initial treatment (n=46)	Corporate hospital	13 (28.3)
	Tertiary care hospital	13 (28.3)
	Local private hospital	8 (17.4)
	Not documented	12 (26.1)
Histopathological types (n=46)	Adenocarcinoma	33(71.7)
	Acinar	5 (10.9)
	Invasive adenocarcinoma	2 (4.3)
	Moderately differentiated adenocarcinoma	1 (2.2)
	Metastatic adenocarcinoma	1 (2.2)
	Not available	4 (8.7)
Gleason score (n=46)	6	9 (19.6)
	7	9 (19.6)
	8	8 (17.4)
	9	9 (19.6)
	NA	11 (23.9)
Disease extent (n=42)	Localized	12 (28.6)
	De novo metastatic	30 (71.4)
Treatment for localized disease (n=12)	Radical prostatectomy	4 (33.3)
	Radical prostatectomy +ADT	3 (25)
	ADT alone	3 (25)
	Surveillance	2 (16.7)
Treatment for metastatic disease (n=36)	Bilateral orchidectomy	20 (55.6)
	LHRH agonist	7 (19.4)
	CAB	6 (16.7)
	Abiraterone	1 (2.8)
	Docetaxel	2 (5.6)
Bone health measures (n=42)	Vitamin D3 + Calcium supplement	2 (4.8)
	Bisphosphonates + Calcium supplement	19 (45.2)
	None	21 (47.6)

Quality indicators for diagnosis

Family history was available for 29 patients (29/46, 63%). A positive family history of any cancer was present in 11 patients (11/29, 37.9%). However, the family history of a specific PC was not documented in the records. A digital rectal examination (DRE) was conducted in five patients (5/46, 10.8%), while prostate-specific antigen (PSA) levels were measured in all 46 patients. However, in five patients, the diagnosis of PC was based only on elevated PSA levels (5/46, 10.8%). The diagnosis was based on a biopsy in 42 patients (42/46, 91.3%), including two patients (2/46, 4.3%) who had biopsy samples collected from an unknown site. Transrectal ultrasound (TRUS)-guided biopsy was conducted in 35 patients (35, 35/46, 76.1%), while transurethral resection (TURP) was conducted in five patients (5/46, 10.9%). These QIs and their compliance in our study are mentioned in Table 2.

**Table 2:** Quality of care indicators of prostate cancer and compliance rate ADT: androgen deprivation therapy; DRE: digital rectal examination; HT: hormone therapy (mentioned separately from ADT as per QI); PC: prostate cancer; PSA: prostate-specific antigen; RT: radiotherapy; TRUS: transrectal ultrasound; TURP: transurethral prostatectomy; WHO: World Health Organization

Domain	Quality care indicator	Numerator	No	Denominator	No	Patients (%)
Diagnosis	Family history of PC documented	Number of patients with a documented family history of PC	0	Number of patients with PC	46	0%
Diagnosis	Proportion of patients with PC and a documented DRE	Number of patients with PC who had a documented DRE	5	Number of patients with PC	46	10.8%
Diagnosis	Proportion of patients with PC and PSA level documented result	Proportion of patients with PC and PSA level documented result	46	Number of patients with PC	46	100%
Diagnosis	Proportion of patients with PC and the diagnosis based only on the PSA result	Number of patients with PC whose diagnosis was based only on the PSA result	5	Number of patients with PC	46	10.8%
Diagnosis	Proportion of patients with PC and the diagnosis based on prostatic biopsy	Number of patients with PC whose diagnosis was based on prostatic biopsy	42	Number of patients with PC	46	91.3%
Pathology	Proportion of patients with PC and the pathology report of the biopsy (TRUS or TURP) including the following characteristics: histologic type according to WHO	Number of patients with PC whose pathology report of the biopsy included the following characteristics: histologic type according to WHO	5	Patients with PC undergoing biopsy (TRUS or TURP)	40	10.8%
Pathology	Proportion of patients with PC and the pathology report of the biopsy (TRUS or TURP) including the following characteristics: Gleason score	Number of patients with PC whose pathology report of the biopsy included the following characteristics: Gleason score	35	Patients with PC undergoing biopsy (TRUS or TURP)	40	87.5 %
Pathology	Proportion of patients with PC and the pathology report of the biopsy (TRUS) including the following characteristics: number of cores	Number of patients with PC and the pathology report of the biopsy (TRUS) including the following characteristics: number of cores	14	Patients with PC undergoing biopsy (TRUS)	35	40%
Pathology	Proportion of patients with PC and the pathology report of the biopsy (TRUS or TURP) including the following characteristics: tumour quantitation (proportion of prostatic tissue involved by tumour/number of positive cores)	Number of patients with PC and the pathology report of the biopsy (TRUS or TURP) including the following characteristics: tumour quantitation (proportion of prostatic tissue involved by tumour/number of positive cores)	9	Patients with PC undergoing biopsy (TRUS or TURP)	40	22.5%
Treatment	Proportion of patients with localized PC received active surveillance	Number of patients with localized PC and receiving only active surveillance	2	Number of patients with localized PC	12	16.7%
Treatment	Proportion of patients with localized PC undergoing radical treatment (radical prostectomy + pelvic lymphadenectomy, RT or brachytherapy + HT)	Number of patients with localized PC undergoing radical treatment (radical prostectomy + pelvic lymphadenectomy, RT or brachytherapy + HT)	10	Number of patients with localized PC	12	83.3%
Treatment	Proportion of patients with localized PC undergoing radical RT + neo adjuvant HT	Number of patients with localized PC undergoing radical RT + neo adjuvant HT	0	Number of patients with localized PC	12	00%
Treatment	Proportion of patients with metastatic PC undergoing HT or bilateral orchiectomy	Number of patients with metastatic PC undergoing HT or bilateral orchiectomy	27	Number of patients with metastatic PC	36	75%
Treatment	Proportion of patients receiving chemotherapy or radiotherapy in the last 30 days of life	Number of patients receiving chemotherapy or radiotherapy in the last 30 days of life	0	Number of patients with PC	42	00%
Treatment	Proportion of patients with PC treated with ADT and received bone health measures	Number of patients with PC treated with ADT and received bone health measures	21	Number of patients with PC	42	50%

Quality indicators for pathology

The pathological type of PC was available for 42 patients, while the data for four patients did not indicate the pathological type of cancer. The majority of patients had conventional adenocarcinoma (33/46, 71.7%) as the histopathological type (Table 1). Based on the World Health Organization (WHO) definition [13], the histological features of the tumor were detailed in the pathology reports (TRUS or TURP) in only five patients (5/40, 12.5%), and the Gleason grading system for histology was followed in 35 patients (35/40, 87.5%).

Out of the 35 patients who had TRUS-guided biopsy, the number of cores was documented in 14 patients (14/35, 40%), and the number of positive cores was mentioned in nine patients (9/35, 25.7%). The proportion of tissue involved was not mentioned in any of the patients who had TURP.

Quality indicators for treatment

Further, the information on QI, as mentioned in Table 2, that refers to the treatment was available in 42 patients only. Localized prostatic cancer was seen in 12 patients (12/42, 28.6%). Among these patients, four patients were treated with radical prostatectomy (4/42, 9.5%), three patients with androgen deprivation therapy (ADT, 3/42, 7.1%), two patients with radiotherapy (RT) + ADT (2/42, 4.7%), and one patient with radical prostatectomy + hormone therapy (HT) (1/42, 2.4%). Among the patients with localized PC, two patients (2/42, 4.7%) had received no further treatment after diagnosis of the primary disease and were on active surveillance.

Thirty patients (30/42, 76.2%) had de novo metastatic disease, and six of the 12 patients (6/12, 50%) who presented with localized disease developed metastasis subsequently. Among patients with metastasis, only 10 patients (10/36, 27.7%) had complete staging before treatment initiation. Only five patients (5/42, 11.9%) had undergone prostate-specific membrane antigen (PSMA) scanning (two for localized disease and three for metastasis). Microsatellite instability status was checked in one patient (1/42, 2.4%), and germline testing for the breast cancer gene (BRCA) was done in one patient (1/42, 2.4%).

Treatment for metastasis included bilateral orchidectomy in 21 patients (21/36, 58.3%), HT in three patients (3/36, 8.3%), and luteinizing hormone-releasing hormone (LHRH) agonist therapy in nine patients (9/36, 25%). One patient each (1/36, 2.7%) had received abiraterone and docetaxel, enzalutamide and docetaxel, as well as docetaxel and abiraterone.

None of the patients had local RT if a low-burden metastatic disease was found. During follow-up, a dual x-ray absorptiometry (DEXA) scan was done in two patients (2/42, 4.7%) only once, and none of the patients had a regular DEXA scan. No specific bone health interventions had been used in 21 patients (21/42, 50%). Bisphosphonate with calcium supplementation was used in 19 patients (19/42, 45.2%), and vitamin D3 with calcium supplementation was used in the other two patients (2/42, 4.8%). Moreover, none of our patients received chemotherapy 30 days before their deaths.

## Discussion

This retrospective study evaluated the quality of PC care. The study made it possible to assess the QoC for PC and identify any potential gaps therein. This study confirms the feasibility of collecting data from medical records to assess the quality of cancer care, as reported earlier [14,15]. Direct information extraction from the original patient records ensures uniform coding and a high level of integrity [6]. The strengths of this study include the selection of the QI developed by the Delphi method by international experts and groups [6,10,11] and the direct extraction of information from the original medical documentation, removing any sample bias.

While a family history of cancer was recorded in more than 50% of our patients and found to be positive in around 30%, a history of PC in the family had not been recorded. Most of our patients received initial treatment in other hospitals, which is indicative of access to healthcare facilities in a predominantly rural, agrarian region. It can be inferred that patients were aware that their disease had treatment options available in hospitals, implying a level of healthcare awareness.

In our study, only five patients reported a DRE. This may indicate poor documentation or reduced reliance on this parameter for clinical diagnosis. Moreover, as per records, 10.8% of patients were given a PC diagnosis only based on PSA.

The diagnosis must be histologically validated to achieve precise grading of the tumor and improved treatment planning [16]. Most of the patients in our study were diagnosed through a biopsy, which conforms with studies conducted in Victoria and South Australia as well as Switzerland [6,17]. For optimal management of PC patients, the information reported in the biopsy pathology reports is of paramount importance. Guidelines published by international organizations like the European Association of Urology suggest that the pathology report of prostatic biopsies should include tumor histology, Gleason score, and quantification of tissue involved in the tumor [18]. The Gleason score was documented in the majority of our patients (85%); however, the histological features and quantification of tissue involvement were less documented. None of the TURP patients and <30% of TRUS patients had quantification of tissue involved by the tumor documented, while histology according to WHO classification was reported in only five patients. This reporting was found to be very low compared to other studies conducted in Switzerland [6].

In our study, most of the patients were diagnosed by a TRUS-guided biopsy (73.9%), and 10.8% had the diagnosis confirmed by TURP. Studies from other countries have also reported similar results [6,17,19]. International guidelines recommend taking a minimum of eight to 12 cores under guidance [18,20,21]. In our study, among the patients with a documented number of cores at TRUS biopsy, only six patients (6/14, 42.8%) had eight or more biopsy cores. As compared to data from other studies where the proportion of patients having more than eight cores taken was more than 70%, this is a much smaller number [6,19,22].

Active surveillance, radical prostatectomy with lymph node dissection, RT, and HT are established methods of treating localized PC [18]. The choice of treatment depends on the risk, patient preference, and physicians’ choice. The number of patients undergoing radical prostatectomy (7/12, 58.3%) was similar to that reported by other studies [6,17]. None of the patients in this group underwent RT for localized disease, probably due to limited access to RT and the desire for quick treatment as opposed to multiple-stage treatment. No referrals for RT were made in this group.

In metastatic PC, treatment aims to prevent the progression of the disease, improve quality of life, and attempt to prolong survival [18]. Either surgical or medical ADT is the treatment of choice for patients with metastatic PC. Recently, the addition of other agents like docetaxel, abiraterone, enzalutamide, or apalutamide has been shown to improve outcomes in patients with high-risk castration-sensitive PC [23]. The use of surgical castration is decreasing despite potential advantages like low cost, equal oncological outcomes, and better non-oncological outcomes. In a recent retrospective study, more men with limited healthcare access underwent orchidectomy [24]. A similar trend was also observed in our study, with orchidectomy being the most frequent intervention (20/36, 55%). Easy access to surgery and lower costs make it a preferable option in countries with limited resources.

Patients with PC are more likely to have increased bone fragility and low bone mineral density [25]. The use of ADT has been associated with bone loss, increasing the risk of skeletal-related events. The prevention of skeletal-related events is an important goal for patients with prostatic cancer [25,26]. Several guidance documents recommend bone mineral density testing before starting ADT [21,27]. In patients with a high risk of fracture, bone-strengthening agents are recommended. In patients with castration-resistant PC and skeletal metastasis, denosumab or zoledronic acid should be used [28,29]. Low utilization of DEXA scans during therapy and bone health interventions is an important lacuna in the management of our patients.

One of the limitations of our study is its small size and the missing data on some patients. Moreover, another limitation, as reported by other studies, is the involvement of multiple specialists in the management of these patients [30]. This is because many patients in our study group received their initial treatment at different institutions. Furthermore, it is not a population-based study, so the findings cannot be generalized. The reasons for the lack of use of RT for localized disease in our group were not studied. The study did not take into account the actual literacy status and educational levels of the population being studied. It is a well-known fact that literacy status impacts access to and utilization of healthcare facilities.

## Conclusions

The current study reveals a lack of compliance for many QoC indicators for PC patients in the study population. The study shows that QI for diagnosis and treatment can be assessed by retrospective chart review, which may help improve therapeutic safety and effectiveness. This will in turn lower variation in care and, thus, enhance patient outcomes. Dissemination of information regarding guidelines for prostate cancer treatment, quality control indicators, and available options for treatment through digital and print media is recommended to plug knowledge gaps that impact healthcare in this region.
